# Structural, Electronic and Vibrational Properties of YAl_3_(BO_3_)_4_

**DOI:** 10.3390/ma13030545

**Published:** 2020-01-23

**Authors:** Aleksandr S. Oreshonkov, Evgenii M. Roginskii, Nikolai P. Shestakov, Irina A. Gudim, Vladislav L. Temerov, Ivan V. Nemtsev, Maxim S. Molokeev, Sergey V. Adichtchev, Alexey M. Pugachev, Yuriy G. Denisenko

**Affiliations:** 1 Laboratory of Molecular Spectroscopy, Kirensky Institute of Physics, Federal Research Center KSC SB RAS, Krasnoyarsk 660036, Russia; nico@iph.krasn.ru; 2 School of Engineering and Construction, Siberian Federal University, Krasnoyarsk 660041, Russia; 3 Laboratory of Spectroscopy of Solid State, Ioffe Institute, St. Petersburg 194021, Russia; e.roginskii@mail.ioffe.ru; 4 Laboratory of Radiospectroscopy and Spintronics, Kirensky Institute of Physics, Federal Research Center KSC SB RAS, Krasnoyarsk 660036, Russia; irinagudim@mail.ru (I.A.G.); bezm@iph.krasn.ru (V.L.T.); 5 Federal Research Center KSC SB RAS, Krasnoyarsk 660036, Russia; ivan_nemtsev@mail.ru; 6 Laboratory of Crystal Physics, Kirensky Institute of Physics, Federal Research Center KSC SB RAS, Krasnoyarsk 660036, Russia; msmolokeev@mail.ru; 7 School of Engineering Physics and Radio Electronics, Siberian Federal University, Krasnoyarsk 660041, Russia; 8 Institute of Automation and Electrometry, Russian Academy of Sciences, Novosibirsk 630090, Russia; adish2@ngs.ru (S.V.A.); apg@iae.nsk.su (A.M.P.); 9 Department of Inorganic and Physical Chemistry, Tyumen State University, Tyumen 625003, Russia; 10 Department of General and Special Chemistry, Industrial University of Tyumen, Tyumen 625000, Russia

**Keywords:** YAl_3_(BO_3_)_4_, huntite-like structure, rare-earth alumoborates, infrared spectra, monoclinic domains

## Abstract

The crystal structure of YAl_3_(BO_3_)_4_ is obtained by Rietveld refinement analysis in the present study. The dynamical properties are studied both theoretically and experimentally. The experimental Raman and Infrared spectra are interpreted using the results of *ab initio* calculations within density functional theory. The phonon band gap in the Infrared spectrum is observed in both trigonal and hypothetical monoclinic structures of YAl_3_(BO_3_)_4_. The electronic band structure is studied theoretically, and the value of the band gap is obtained. It was found that the YAl_3_(BO_3_)_4_ is an indirect band gap dielectric material.

## 1. Introduction

**Figure 1:**
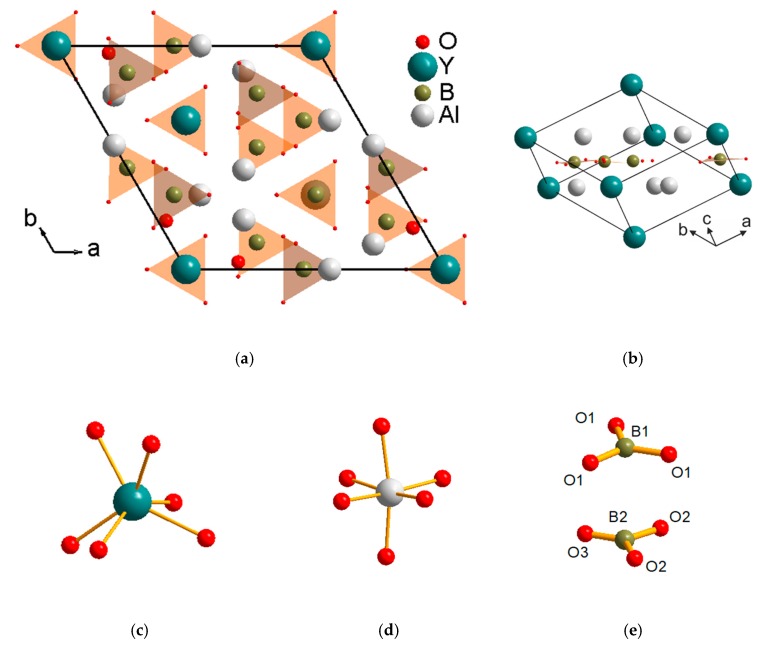
Projection of the YAl_3_(BO_3_)_4_ unit cell on the basal plane (**a**) and it’s primitive cell (**b**). Basic structural units: (**c**) YO_6_, (**d**) AlO_6_ and (**e**) BO_3_.

During the past decades, borate crystals have become of extensive interest due to a wide variety of structures [1]. Borates are transparent in a wide spectral range, and possess a good chemical and mechanical stability. The optical properties of borate crystals depend of their crystal structure which can be constructed from [BO_3_]^3−^ and [BO_4_]^5−^ ions [2,3]. Recently, the borates with huntite structure (CaMg_3_(CO_3_)_4_, *R*32 space group) are the subject of considerable interest due to valuable magnetoelectric [4,5] and spectroscopic [6,7,8] properties which are promising for technical applications. The general formula of the huntite-like borates is *ReM*_3_(BO_3_)_4_ where Re = lanthanide, M = Al, Sc, Cr, Fe, Ga. The YAl_3_(BO_3_)_4_ (YAB) was synthesized for the first time in 1960s [9,10], and the relative high hardness (Mohs hardness 7.5) and chemical stability were discovered at the same time [9]. The crystallographic and primitive unit cells of YAl_3_(BO_3_)_4_ are shown in Figure 1a,b correspondingly. The yttrium ions occupy the position with the *D_3_*(*32*) site symmetry in the crystal lattice and have six-fold oxygen coordination (Figure 1c). The nearest-neighbor environment of aluminum ions (*C_2_*(*2*) site) forms octahedral oxygen coordination (Figure 1d). The boron ions are surrounded by three oxygen atoms which form triangles and occupy the *D_3_*(*32*) and *C_2_*(*2*) positions. One of them composed by atoms labeled as B1 and O1, and the other one contains B2, O2 and O3 atoms (Figure 1e) [11].

Recently, many rare-earth [12,13,14,15,16,17] and rare-earth doped compounds [18,19,20,21,22,23,24] have been evaluated as phosphors. In case of YAl_3_(BO_3_)_4_ host, the rare-earth ions occupy *D*_3_(*32*) site in the structure (the center of a distorted trigonal prism) and substitute a part of Y^3+^ ions.

The Eu^3+^ ions doped into the YAl_3_(BO_3_)_4_ lattice pursuing a red phosphor with good colorimetric features for display panels applications [25].

The reddish-orange emission has been obtained from the Sm^3+^ doped YAl_3_(BO_3_)_4_ polycrystalline samples under near UV excitation [26]. The intense blue luminescence under UV excitation is observed in the Tm^3+^ doped YAl_3_(BO_3_)_4_ samples [27]. The Er^3+^/Yb^3+^ co-doped YAl_3_(BO_3_)_4_ crystal is a promising material for 1.5 μm lasers.

The emission with output power within the range of 0.8–1 W is obtained at different wavelengths: 1602, 1550, 1543 and 1520 nm [28]. The spectroscopic properties of Er,Yb:YAl_3_(BO_3_)_4_ crystals have been investigated at both ambient and high temperature (300–800K) conditions [29]. It has been shown that the high-performance eye-safe 1.55 μm microchip laser can be fabricated by the tightly pressurization of two sapphire crystals with high thermal conductivity and the Er:Yb:YAl_3_(BO_3_)_4_ laser crystal between them [30,31]. The narrow lines attributed to the Mn^4+^ ions (682, 684 and 686 nm) are observed in the luminescence spectra of YAB:Mn under 514.5 nm excitation [32].

It was previously established that the rare-earth borates represent three polymorphic modifications: the huntite structure (space group *R*32) and two monoclinic structures with *C*2/*c* and *C*2 space groups [33]. However, the weak bands of possible monoclinic (*C*2/*c*) polytype of *ReM*_3_-borates (*Re* is Nd, Gd and Y; *M* is Al, Ga, Cr, and Fe) have been found in the Infrared spectrum of samples with huntite structure [34]. Co-existence of trigonal and monoclinic phases can produce, for example, the effect of local stresses or decreasing of the nonlinear properties. The variation from non-centrosymmetric (*R*32) to centrosymmetric (C2/c) structure will affect to nonlinear optical and magnetoelectric properties.

The structural characterization of YAl_3_(BO3)_4_ host lattice is mainly related to X-ray diffraction [25,35,36,37]. The main purpose of this study is to study vibrational spectra of YAB and reveal or exclude a possible portion existence of the monoclinic (*C*2/*c*) phase in trigonal (*R*32) YAl_3_(BO_3_)_4_ lattice. The results of this work can be used in part to study vibrational properties of a set of *ReM*_3_(BO_3_)_4_ family members. The investigation of electronic, structural and vibrational properties of YAB is based on performing *ab initio* calculations in the framework of density functional theory calculations and a group of experimental techniques: Infrared, energy-dispersive X-ray and Raman spectroscopy, and X-ray diffraction analysis.

## 2. Materials and Methods

### 2.1. Synthesis

Single crystals of YAl_3_(BO_3_)_4_ have been grown from the {88% mass [Bi_2_Mo_3_O_12_ + 2B_2_O_3_ + 0.5Li_2_MoO_4_] + 12 wt % YAl_3_(BO_3_)_4_} solution-melt [38]. The saturation temperature of this solution-melt was determined as *T*_sat_ = 980 °C. The concentration (*n*) dependence of the saturation temperature had a slope *dT*_sat_/*dn* =15 °C/wt %.

The solution-melt of 150 g total weight was prepared in a cylindrical platinum crucible (*D* = 50 mm, *h* = 60 mm) by sequential melting of oxides (Bi_2_O_3_ + MoO_3_), B_2_O_3_, Y_2_O_3_, (Li_2_CO_3_ + MoO_3_) at T = 1000−1100 °C. The saturation temperature was defined with accurate to within ± 2 °C with the use of spontaneous probe crystals grown previously.

Group method was used to growth crystals. Four seeds with size ~ 1 mm^3^ were attached to the rod crystal holder. The initial supercooling was corresponded to the middle of the metastability zone and footed up to 10 °C. After this, the temperature of the solution-melt was reduced according to the program with an increasing rate of 1–3 °C/day. The rate of crystal growth did not exceed 0.5 mm/24 h. The rod crystal holder was rotated reversibly with a 1-min period. After the growth was finished, the rod crystal holder had been raised above the solution-melt and the furnace was cooled to room temperature with the rate of 100 °C/h. The YAl_3_(BO_3_)_4_ crystals with dimensions of 6–10 mm and a total mass of 10 g were obtained in the crystallization temperature interval of 17 °C.

### 2.2. Experimental

The Infrared (IR) absorption spectrum was recorded with a Fourier-transform spectrometer VERTEX 70 V (Bruker, Billerica, MA, USA) in the spectral range from 400 to 1600 cm^−1^ with spectra resolution 4 cm^−1^. The spectrum was taken from a tablet sample shaped as about 0.4 mm thick tablet of 13 mm in diameter and a weight of 0.15 g. The tablet was prepared as follows: 0.00338 g of YAl_3_(BO_3_)_4_ was thoroughly ground with 0.20 g of KBr. The Globar was used for light source, and it was equipped with a KBr wide beamsplitter and RT-DLaTGS as a detector (Bruker, Billerica, MA, USA).

The morphology of the sample was characterized with a Tabletop Microscope TM3000 (Hitachi, Tokyo, Japan) equipped with an EDX X-Flash 430 (Bruker, Billerica, MA, USA) with an acceleration voltage of 15 kV. Its chemical composition (mapping) was investigated with a detection time of 100 s. To avoid a surface charge-up as well as to improve an imaging quality of the SEM (scanning electron microscope) micrographs a thin platinum film was deposited with a sputter coater K575XD (Emitech, Houston, TX, USA) with 3 cycles. The average coating time was of the order of 1 min using a plasma current of 10 mA.

The X-Ray powder diffraction data of YAl_3_(BO_3_)_4_ was collected at room temperature with a Bruker D8 ADVANCE powder diffractometer (Cu-Kα radiation, 40 kV, 40 Ma, Bruker, Billerica, MA, USA) and linear VANTEC detector. The step size of 2θ was 0.016°, and the counting time was 1 s per step. The intensities from single crystal YAl_3_(BO_3_)_4_ of 0.2 × 0.1 × 0.1 mm dimensions were collected at 296 K using the SMART APEXII X-ray four-circle single crystal diffractometer (Bruker) equipped with a CCD-detector, graphite monochromator and Mo Kα radiation source. The cell parameters were refined by 1525 reflections. The X-ray data from crystal were measured with the exposure time of 10 s on each frame. Crystal rotated along ω-axis by 0.5° at the fixed φ angle and the ω value was increased from 0° to 182°. Totally the 364 frames were measured at each fixed φ equal to 0°, 120° and 240°. After that, the program APEXII from Bruker integrated the intensities of reflections. Space group *R*32 was defined by the analysis of extinction rules and intensity statistics obtained from all reflections. Multiscan absorption correction of reflection intensities was performed by APEXII software (Bruker, Billerica, MA, USA). Then, the intensities of equivalent reflections were averaged.

The Raman spectra study of the single crystal sample was carried out at room temperature in a back-scattering geometry. The laser irradiation of solid-state laser (532.1 nm, Spectra-Physics Millennia) was used for the Raman experiment after passing a monochromator to suppress parasitic laser lines. A triple-grating spectrometer TriVista 777 (Princeton Instruments, Acton, USA) was used for the Raman scattering registration in a frequency range from 18 to 1700 cm^−1^ with spectral resolution ∼1 cm^−1^. For the wavelength calibration of the spectrometer, a neon-discharge lamp was used.

### 2.3. Calculation Details

Density functional (DFT) calculations were performed using the plane–wave pseudopotential method as implemented in the CASTEP code [39]. The structural parameters of YAl_3_(BO_3_)_4_ were fully optimized using the local density approximation (LDA) provided by the Perdew and Zunger [40] parameterization of the numerical results of Ceperley and Alder (CA-PZ) [41]. The calculations were performed using norm conserving pseudopotentials with 2s^2^2p^1^ electrons for B, 2s^2^2p^4^ electrons for O, 3s^2^3p^1^ electrons for Al, and 4d^1^5s^2^ electrons for Y atom treated as a valence ones. The tolerance in a self-consistent field (SCF) procedure was set to be 5.0 × 10^−8^ eV/atom and total energy was corrected for a finite basis set. The convergence tolerance for geometry optimization was selected with the differences in maximal force and stress tensor within 0.0001 eVÅ^−1^ and 0.01 GPa correspondingly. The energy cutoff of 900 eV was used with 4 × 4 × 4 sampling of the Brillouin zone (BZ) using the Monkhorst–Pack scheme [42]. The phonon spectra at the Γ-point of the BZ was calculated within density functional perturbation theory and finite displacement method [43,44] based on the crystal system type. The dispersion of phonon branches along high symmetry directions of the BZ was calculated using a linear response formalism [45].

## 3. Results and Discussion

The main information about crystal data, data collection and refinement are reported in Table 1. The structure was solved by the direct methods using package SHELXS and refined in the anisotropic approach for non-boron atoms using SHELXL program [46]. The structural tests for the presence of missing symmetry elements and possible voids were produced using the PLATON program [47]. The main crystal data are shown in Table 1. The coordinates of atoms are reported in Table S1 and main bond lengths are shown in Table S2 of supplementary materials.

**Figure 2:**
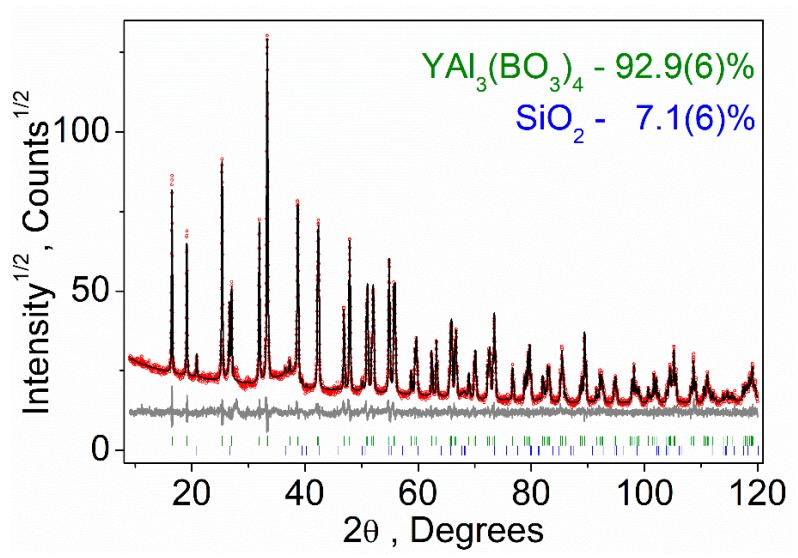
Difference Rietveld plot of YAl_3_(BO_4_)_3_ with small amount of SiO_2_ impurity, which was appeared after grinding in agate mortar.

Almost all peaks of the powder X-ray diffraction pattern, besides impurity SiO_2_ peaks, were indexed by trigonal cell (*R*32) with parameters close to the previously published YAl_3_(BO_3_)_4_ [10] and identical to parameters of investigated single crystal (see Table 1). The SiO_2_ impurity was appeared after grinding YAl_3_(BO_3_)_4_ in the agate mortar, while the initial YAl_3_(BO_3_)_4_ bulk material was pure. The structure obtained from single crystal examination was taken as a starting model for multiphase Rietveld refinement method [48] which was performed using TOPAS 4.2 [49] software package. Refinement was stable and gave low *R*-factors (Table 2, Figure 2). Coordinates of atoms and main bond lengths are presented in Tables S3 and S4 of supplementary materials, respectively. The crystallographic data are deposited in Cambridge Crystallographic Data Centre (CCDC #1960228). The data can be downloaded from the site (www.ccdc.cam.ac.uk/data_request/cif).

Next, obtained structural parameters were taken as initial for the *ab initio* geometry optimization included the unit cell parameters and atomic positions. The optimized structure is consistent with experimental data as shown in Table S5 of supplementary materials.

**Figure 3:**
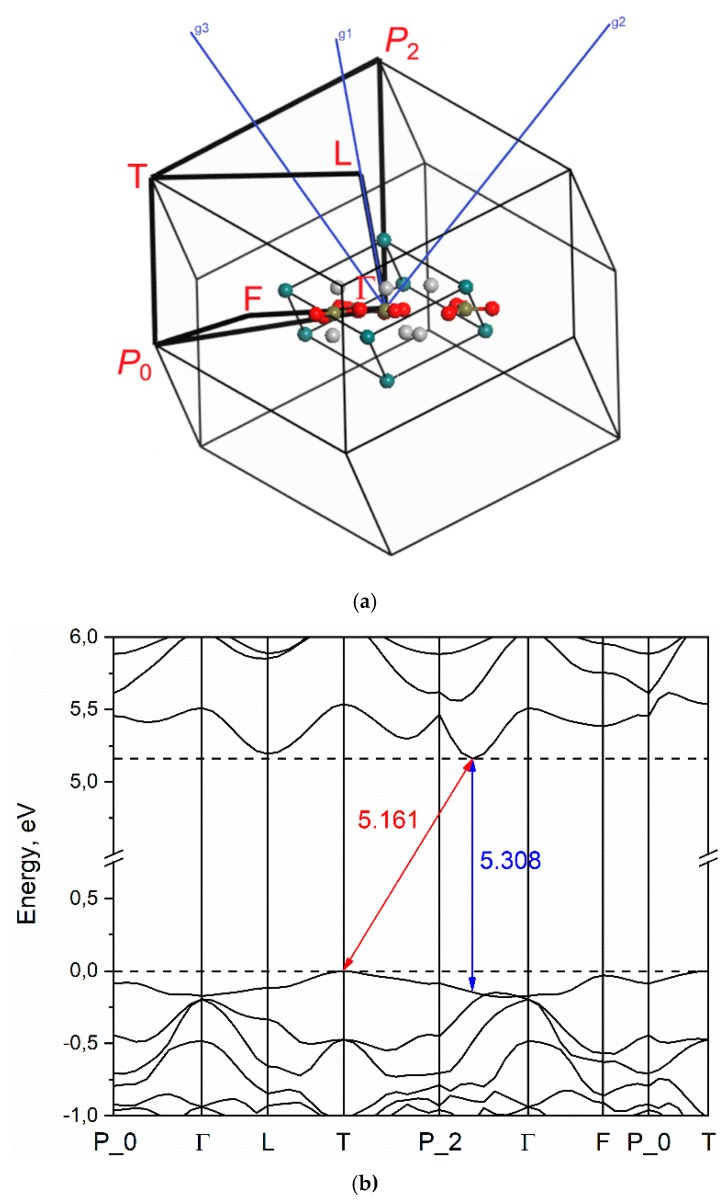
Brillouin zone of the YAl_3_(BO_3_)_4_ rhombohedral lattice (**a**) and electronic band structure (**b**).

The high-symmetry points of the BZ are selected as *P*_0_–Γ–L–T–*P*_2_–Γ–F–*P*_0_–T for calculation of the YAB band structure. The coordinates of the special points of the Brillouin zone are: *P*_0_(0.298, −0.702, 0.298), Γ(0, 0, 0), L(0.5, 0, 0), T(0.5, −0.5, 0.5), *P*_2_(0.301, 0.301, 0.301), F(0.5, −0.5, 0), T(0.5, −0.5, 0.5) [50,51] and points are shown in Figure 3a. The results of the calculation of the yttrium aluminum borate band structure are presented in Figure 3b.

The value of the band gap is defined as the difference between the conduction band minimum (CBM) and the valence band maximum (VBM). It is found that the VBM is well localized in the vicinity of the T-point and the CBM is located between the *P*_2_ and Γ points. The band gap value for indirect electronic transitions is *E*^i^_g_ = 5.161 eV. The lowest energy direct transition is found in the vicinity of *P*_2_ point of the BZ (the point in the *P*_2_→Γ direction), also the direct transition with approximately the same energy is obtained in L-point of the BZ (see Figure 3b). The value of the direct bandgap is equal to *E*^d^_g_ = 5.308 eV. The obtained value of band gap is underestimated compared with the experiment value of 5.7 eV [52] which can be explained as a systematic DFT problem due to well-known band gap underestimation problem [53].

The hybrid functional HSE06 [54] method was developed to improve the accuracy of the band structure calculations. The value of bandgap *E*^i^_g_ =7.2 eV was calculated using the hybrid functional method. The obtained value is significantly overestimate the experimental value reported in [52]. There is no experimental absorption spectrum presented in the paper [52] only the theoretical one, therefore no evidence that the value of the band gap is correctly extracted (for example extrapolated with Kubelka–Munk equation [55]) from experimental data. We suggest the new experiments on the determination of the band gap would clarify more accurate value.

According to the Y. Wang et. al. [56] the VBM is at the M point and the CBM is at A point of the BZ (crystallographic hexagonal unitcell); calculated band structure of YAB is presented in work of M.G. Brik et. al. [57] but the nature of electronic transitions is not discussed; according to the work of R. He [58] the YAB is a material with a direct band gap (Γ-point) but it is noteworthy that the band structure was calculated along two paths in BZ only, therefore the bandstructure in [58] is not complete. The direct transition in Γ-point was also obtained in Ali H. Reshak’s work [59], but a significantly lower value of the cutoff energy was used in the calculations, therefore the basis set is not complete. No experimental investigation of the band structure was found, hence if the YAB is a direct transition crystal or not is an open question. The hexagonal unitcell is three times bigger than the primitive rhombohedral one, hence the volume of the Brillouin zone is three times lower. We perform calculations using rhombohedral unit cell and along all with known high-symmetry directions, therefore the results obtained in recent research more straightforward.

**Figure 4:**
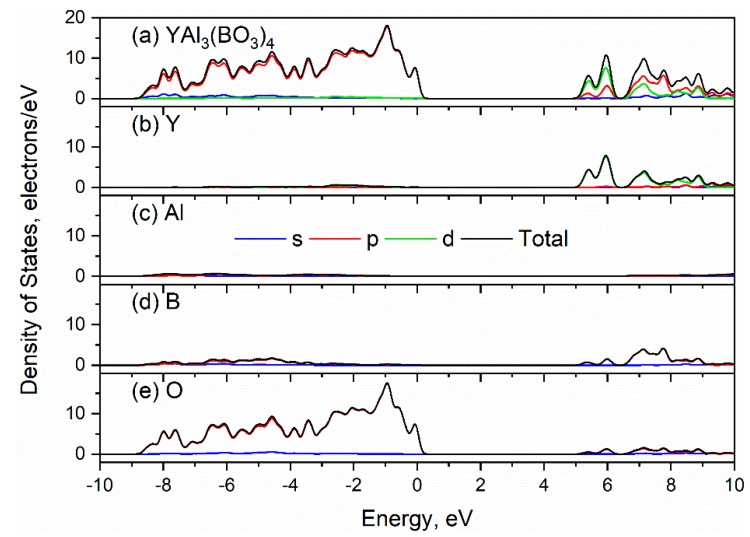
Total (**a**) and partial density of states (**b**), (**c**), (**d**), (**e**) of YAl_3_(BO_3_)_4._

The total and partial density of states of the YAl_3_(BO_3_)_4_ structure are plotted in Figure 4 and Figure S1 of supplementary materials. As a result of the figure analysis, one can find that the valence band maximum is formed mostly by p-electrons of oxygen atoms while the conduction band minimum is constructed mostly by d-electrons of yttrium, p-electrons of boron and p-electrons of oxygen atoms. It clearly seen, that the contribution of the aluminum ions to the total DOS significantly less than other ions. Therefore, one can assume the Al and Y atoms of YAl_3_(BO_3_)_4_ crystal are found to be in (III) valence state.

**Figure 5:**
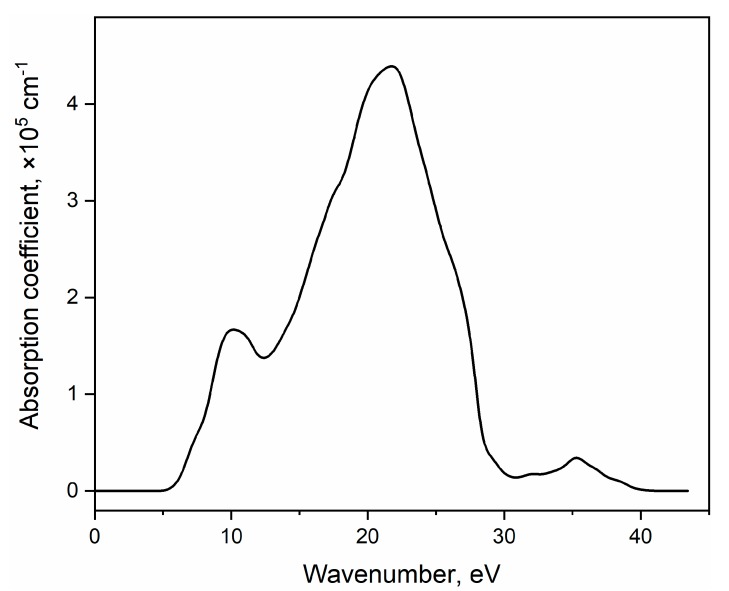
The calculated YAl_3_(BO_3_)_4_ absorption coefficient versus photon energy.

The absorption coefficient calculated by LDA using a scissor operator (the difference between the theoretical and experimental [52] band gap values) equal to 0.539 eV is plotted in Figure 5. From the partial density of states analysis (Figure 4 and Figure S1), it follows that the first peak the spectrum is associated with electronic transitions mainly from the 2p orbitals of the O atom to the 4d orbitals of Y atoms.

**Figure 6:**
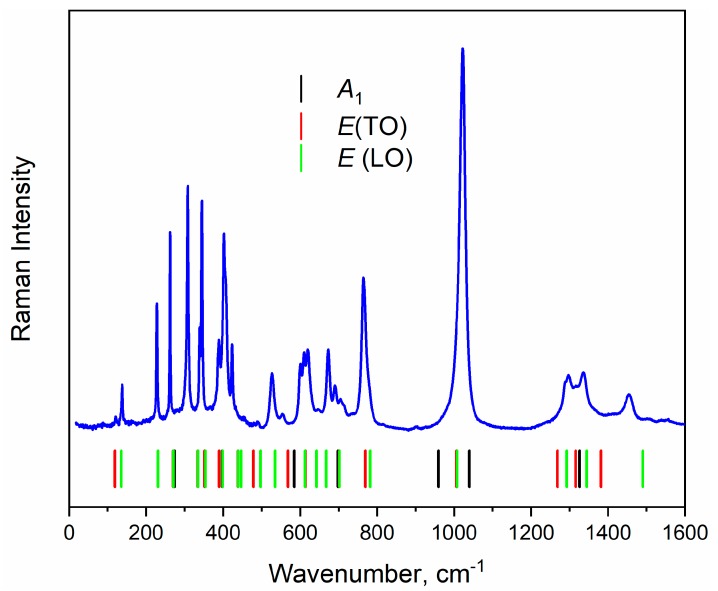
Raman spectra of YAl_3_(BO_3_)_4_ recorded at 532.1 nm. Vertical lines show the positions of calculated Raman-active bands.

**Figure 7:**
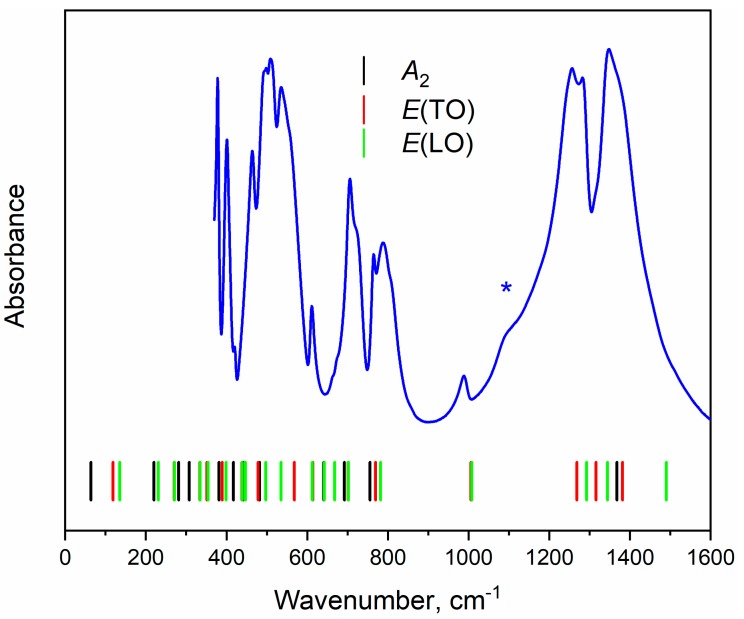
Infrared absorption spectra of YAl_3_(BO_3_)_4_ in Mid-IR sub region, and the artefact is shown with an asterisk. Vertical lines show the positions of calculated IR-active bands.

The nonpolarized Raman and Infrared spectra are shown in Figure 6 and Figure 7 correspondingly. Polarized Raman spectra are plotted in Figures S2 and S3 and simulated Raman spectra for specific Raman tensor components are shown in Figures S2–S4 of supplementary materials. The mechanical representation for the YAl_3_(BO_3_)_4_ at Brillouin zone center is Γ_vibr_ = 7*A*_1_ + 13*A*_2_ + 20*E* [60] where Raman active modes are Γ_Raman_ = 7*A*_1_ + 19*E*, and infrared active modes are Γ_Infrared_ = 12*A*_2_ + 19E. The acoustic modes are Γ_Acoustic_ = *A*_2_ + *E*. The *A* and *E* letters correspond to nondegenerate and doubly degenerate vibrations correspondingly. The *E* modes are polar and active as in Raman as in IR spectra.

The symmetry of [BO_3_]^3−^ ions and type of vibrations was described by Nakamoto [61]. It was found that the point group of [BO_3_]^3−^ is *D*_3*h*_ and the decomposition of vibrational spectra by irreducible representations is as follows: *A*_1_’ + 2*A*_2_” + 3*E*’ + *A*_2_’ + *E*”. The mode *ν*_1_ (*A*_1_’) is a symmetric stretching vibration, *ν*_2_ (*A*_2_”) is off-plane deformational vibration, *ν*_3_ and *ν*_4_ (*E*’) is in-plane deformational vibration. Normal modes of vibrations of [BO_3_]^3−^ ions discussed above are presented in Figure S5 of supplementary materials. Finally, there are three translational vibrations, one (along high symmetry axis) with symmetry *A*_2_” and two *E*”, and three rotational vibrations *A*_2_’ and *E*”. The boron atoms in the host YAl_3_(BO_3_)_4_ unit cell is found to take two Wyckoff positions, namely *3b* (site symmetry *D*_3_) and *9e* (site symmetry *C*_2_). The correlation diagram of internal vibrations between the free [BO_3_]^3−^ ions with *D*_3*h*_ symmetry, its site symmetries (*D*_3_ and *C*_2_) and factor group symmetry *D*_3_ of host unit cell is shown in Table 3. The calculated phonon frequencies of the YAl_3_(BO_3_)_4_ are given in Table S6.

According to Table 3 and Table S6 in supplementary materials, the Raman spectrum around 1000 cm^−1^ should consist of 2*A*_1_ + *E*(TO) + *E*(LO) modes and these bands related to symmetric stretching of [BO_3_]^3−^ ions. The experimental B1–O1 bond length is equal to 1.396 Å and bond lengths are 1.389 and 1.382 for B2–O2 and B2–O3 correspondingly. The B–O bond lengths values obtained after geometry optimization are 1.373, 1.367 and 1.351 for B–O1, B–O2 and B–O3 correspondingly. The frequency of the [BO_3_]^3−^ symmetric stretching vibration is higher in case of B2O_3_ than of B1O_3_, thus we can see that the shorter B–O bonds give higher vibrational frequencies. The range of Raman spectrum 1260–1430 cm^−1^ is related to antisymmetric stretching of the BO_3_ planar group and should consist of *A*_1_ + 3*E*(TO) + 3*E*(LO) bands. The spectral bands in this range are overlapped, the only one single Raman line is at 1453 cm^−1^ and corresponds to *E* (LO) vibrational mode.

The Infrared-active stretching vibrations of [BO_3_]^3−^ ions predicted for YAl_3_(BO_3_)_4_ using factor group theoretical analysis are *A*_2_ + 4*E*(TO) + 4*E*(LO), Table 3. According to the results of calculations, these vibrations should be in the range of 1000–1500 cm^−1^. The spectral band at 990 cm^–1^ corresponds to *E*(TO) + *E*(LO) modes and shift in band positions due to TO-LO splitting is insignificant. The spectral range of 1250–1500 cm^−1^ should consist of remain modes (*A*_2_ + 3*E*(TO) + 3*E*(LO)). However, the decomposition of experimental spectra in the range of stretching vibration revealed extra bands that is not in accordance with calculations. The clearly seen extra band around 1100 cm^−1^ is marked with an asterisk in Figure 7. The typical Infrared spectra of huntite-like ReM_3_(BO_3_)_4_ (Re = Y, rare-earth element, M = Al, Ga, Fe, Cr) compounds with noncentrosymmetric trigonal structure (*R*32 space group) should contain an empty gap in the range 1050–1200 cm^−1^ [62,63,64]. However, as discussed earlier [65,66,67], the borates with large rare-earth elements can form not only trigonal but also monoclinic structures depending on the growth conditions. Some extra bands (in comparison with trigonal structure) were observed in the range of 1050–1200 cm^−1^ and these bands assigned to ν_3_ vibrations of BO_3_^3−^ ions [67,68]. The presence of the band at 1100 cm^−1^ has been attributed to the presence of monoclinically ordered domains incorporated into the trigonal structure [68]. Recently, the monoclinic domains have been observed directly in EuAl_3_(BO_3_)_4_ by means of high resolution transmission electron microscopy (HRTEM) investigations [69] and extra bands in Infrared spectra have been also observed. However, the group of extra peaks (in comparison with observed for YAl_3_(BO_3_)_4_) clearly seen in Infrared spectra of EuAl_3_(BO_3_)_4_ in the area of stretching vibrations of [BO_3_]^3−^ ions at 872, 931, 980 and 1050 cm^−1^.

**Figure 8:**
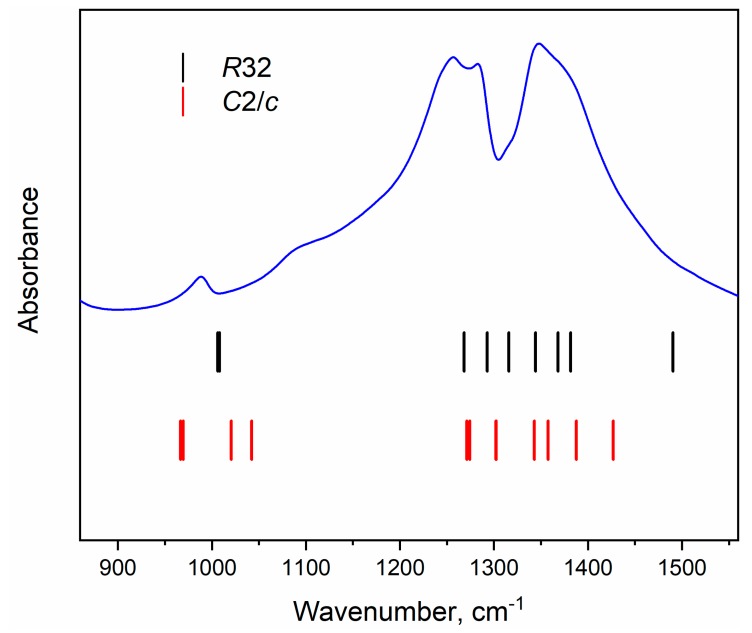
The IR absorption spectra of YAl_3_(BO_3_)_4_ in the range of stretching vibration of BO_3_ triangles in comparison with calculated wavenumbers (vertical lines) of IR-active vibrations in trigonal (R32) and hypothetical monoclinic (C2/c) structures.

We have carried out first principles calculations of the vibrational spectrum of YAl_3_(BO_3_)_4_ isostructural to published monoclinic structure of β-NdAl_3_(BO_3_)_4_ [70]. The comparison of experimental Infrared spectra in the range of [BO_3_]^3−^ stretching vibrations (950–1500 cm^−1^) and calculated wavenumber values are shown in Figure 8. According to the factor group analysis (Table 4) and results of *ab initio* calculations, one can conclude that two crystallographically independent BO_3_^3−^ ions should produce four spectral bands in the range of ν_1_ vibrations, empty gap between 1050–1250 cm^−1^ and eight spectral bands related to ν_3_ vibrations in the range of 1250–1450 cm^−1^. Similar characteristics of Infrared spectra observed only for EuAl_3_(BO_3_)_4_ [69] but not for other *ReM*_3_(BO_3_)_4_ [34,63,66,67,68]. In case of Sm^3+^ doped YAl_3_(BO_3_)_4_ several bands have been found at 869, 918 and 1064 cm^−1^ but X-ray diffraction diffractograms do not contain reflexes related to monoclinic phases [71].

**Figure 9:**
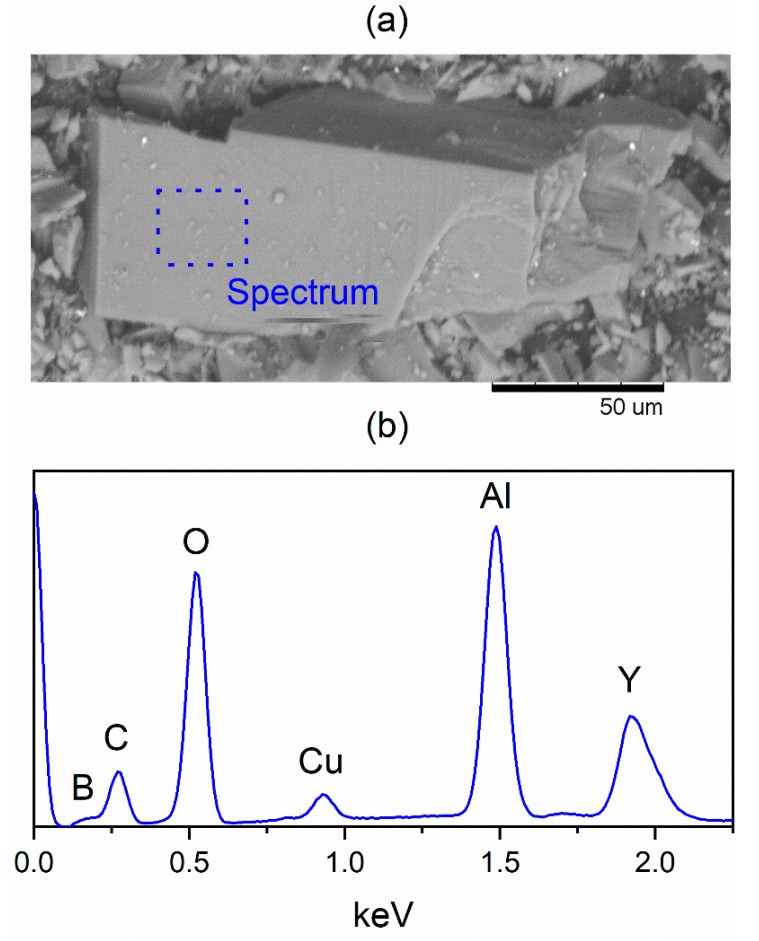
(**a**) Micrograph and (**b**) energy-dispersive X-Ray (EDX) spectrum of YAl_3_(BO_3_)_4._

The energy-dispersive X-ray (EDX) microanalysis was used to study the elemental composition of YAl_3_(BO_3_)_4_ crystals (Figure 9a). The component spectrum (Figure 9b) contains peaks of boron, oxygen, aluminum, yttrium, carbon and copper. The last one is related to the copper substrate. It is well known that EDS, in contrast to Auger spectroscopy, is a more accurate method for heavy elements (atomic number > 33). That is why the carbon quantity in the spectrum is overestimated. Moreover, there are a lot of carbon contaminants in any SEM chambers, that affect carbon quantity in spectra. In addition, a carbon conductive double-coated tape was used to mount the sample to operate in SEM. We cannot exclude from the discussion the molybdenum because molybdenum oxides are part of the synthesis components, however, molybdenum is not found.

On the other hand, the frequency of Si–O–Si stretching vibration in SiO_2_ (1100 cm^–1^) [72] perfectly matches the frequency of extra band in Infrared spectra of YAl_3_(BO_3_)_4_ obtained in Infrared spectrum (Figure 7). Therefore, the nature of the spectra band is an open question.

## 4. Conclusions

As a result of this work, we can conclude that the investigated sample of YAl_3_(BO_3_)_4_ belongs to a group of borates with huntite structure. The calculated band structure shows YAl_3_(BO_3_)_4_ to be indirect band gap dielectric with *E*^i^_g_ = 5.161 eV. The value of the direct bandgap is equal to *E*^d^_g_ = 5.308 eV, which is close to the value of indirect transition. It was clearly shown that the structural analysis of YAl_3_(BO_3_)_4_ should be done on a framework of several methods, for example, a combination of computational, diffraction and spectroscopic methods. It was obtained that the excess bands in the range of 1050–1200 cm^−1^ of the Infrared spectrum do not correspond to the possible monoclinic phase of YAl_3_(BO_3_)_4_ suggested by Dobretsova et al. [68].

Based on current research results the future activities can be aimed to obtain the vibrational spectra of monoclinic domains in ReAl_3_(BO_3_)_4_, where Re = Y or rare-earth elements (with the exception of EuAl_3_(BO_3_)_4_ [69]), or vibrational spectra of *Re*Al_3_(BO_3_)_4_ with totally monoclinic structure.

## Figures and Tables

**Table 1:** Main parameters of processing and refinement of the YAl_3_(BO_3_)_4_ single crystal.

YAl_3_(BO_4_)_3_ Single Crystal	
Molecular weight	405.09
Temperature (K)	296
Space group, *Z*	*R*32, 3
*a* (Å)	9.2863 (10)
*c* (Å)	7.2311 (8)
*V* (Å^3^)	540.03 (13)
ρ_calc_ (g/cm^3^)	3.737
μ (mm^−1^)	8.557
Reflections measured	1525
Reflections independent	302
Reflections with *F* > 4σ(*F*)	302
2θ_max_ (°)	58.49
*h*, *k*, *l* - limits	−12 ≤ *h* ≤ 12; −12 ≤ *k* ≤ 12; −9 ≤ *l* ≤ 9
*R*_int_	0.0336
*Refinement Results*	
The weighed refinement of *F*^2^	*w* = 1/[σ^2^(*F*_o_^2^) + (0.0102*P*)^2^] where *P* = max(*F*_o_^2^ + 2*F*_c_^2^)/3
Number of refinement parameters	33
*R*1 [*F*_o_ > 4σ(*F*_o_)]	0.0153
*wR*2	0.0384
*Goof*	1.164
∆ρ_max_ (e/Å^3^)	0.57
∆ρ_min_ (e/Å^3^)	−0.38
(∆/*σ*)_max_	<0.001
Extinction coefficient (SHELXL 2014/7)	0.072 (5)

**Table 2:** Main parameters of processing and refinement of the YAl_3_(BO_4_)_3_ powder.

YAl_3_(BO_4_)_3_ Powder	
Sp.Gr., Z	*R*32, 3
*a*, Å	9.28485 (7)
*c*, Å	7.23005 (8)
*V*, Å^3^	539.79 (1)
*Z*	3
*2θ*-interval, °	9–120
*R_wp_*, %	7.05
*R_p_*, %	5.42
*R_exp_*, %	4.19
*χ^2^*	1.68

**Table 3:** Correlation diagram of internal vibrations of the BO_3_^3−^ in the YAB.

Free ion Symmetry	Site Symmetry	Factor Group Symmetry	Site Symmetry	Factor Group Symmetry
*D*_3*h*_	*D*_3_	*D*_3_	*C*_2_	*D*_3_
ν_1_, *A*′_1_	*A*_1_	*A*_1_	*A*	*A*_1_ + *E*
ν_2_, *A*″_2_	*A*_2_	*A*_2_	*B*	*A*_2_ + *E*
ν_3_, *E*′	E	E	*A* + *B*	*A*_1_ + *A*_2_ + 2*E*
ν_4_, *E*′	E	E	*A* + *B*	*A*_1_ + *A*_2_ + 2*E*

**Table 4:** Correlation diagram of internal vibrations of the BO_3_^3−^ in case of hypothetical monoclinic structure of the YAB.

Free ion Symmetry	Site Symmetry	Factor Group Symmetry
*D*_3*h*_	C_1_	C^6^_2*h*_
ν_1_, *A*′_1_	*A*	*A_g_ + A_u_+ B_g_ + B_u_*
ν_2_, *A*″_2_	*A*	*A_g_ + A_u_ + B_g_ + B_u_*
ν_3_, *E*′	2*A*	2*A_g_ +* 2*A_u_ +* 2*B_g_ +* 2*B_u_*
ν_4_, *E*′	2*A*	2*A_g_ +* 2*A_u_ +* 2*B_g_ +* 2*B_u_*

## Supplementary Materials

The following are available online at https://www.mdpi.com/1996-1944/13/3/545/s1, Table S1: Fractional atomic coordinates and isotropic or equivalent isotropic displacement parameters (Å2) of YAl_3_(BO_3_)_4_ single crystal, Table S2: The main bond lengths (Å) of YAl_3_(BO_3_)_4_ single crystal, Table S3: Fractional atomic coordinates and isotropic displacement parameters (Å2) of YAl_3_(BO_4_)_3_ powder, Table S4: Main bond lengths (Å) of YAl_3_(BO_4_)_3_ powder, Table S5: Calculated optimized lattice parameters and atomic positions of YAl_3_(BO_3_)_4_ in comparison with the experimental data, Table S6: Calculated and experimental phonon frequencies (cm^−1^) of YAl_3_(BO_3_)_4_ together with proposed assignments. Notations: ss – symmetric stretching, as – antisymmetric stretching, π – out-of-plane bending, δ – in-plane bending, libr. – librations, tr – translations, Figure S1: Total (a) and partial density of states (b), (c), (d), (e) of YAl_3_(BO_3_)_4_, Figure S2: Polarized Raman spectrum of YAB single crystal obtained from the -z(xx)z orientation, Figure S3: Polarized Raman spectrum of YAB single crystal obtained from the -*z*(*xy*)*z* orientation, Figure S4: Calculated Raman spectra of YAB in the -*x*(*zz*)*x*, -*x*(*yz*)*x* and -*x*(*yy*)*x* polarizations, Figure S5: Normal modes of vibration of [BO_3_]^3−^ ions: (a) ν_1_ symmetric stretching, (b) ν_2_ out-of-plane bending, (c) ν_3_ antisymmetric stretching, (d) ν_4_ in-plane bending.
