# Supplementary material for: Structural, Electronic and Vibrational Properties of YAl3(BO3)4
Source: Materials (Basel). 2020 Jan 23;13(3):545. doi: 10.3390/ma13030545 (PMC7040588; doi:10.3390/ma13030545)
Supplement: Supplementary file 1 [file materials-13-00545-s001.pdf]

materials

MDPI

Article

Structural, Electronic and Vibrational Properties of $YAl_3(BO_3)_4$

Aleksandr S. Oreshonkov [1,2,*], Evgenii M. Roginskii [3], Nikolai P. Shestakov [1], Irina A. Gudim [4], Vladislav L. Temerov [4], Ivan V. Nemtsev [5], Maxim S. Molokeev [6,7], Sergey V. Adichtchev [8], Alexey M. Pugachev [8] and Yuriy G. Denisenko [9,10]

1 Laboratory of Molecular Spectroscopy, Kirensky Institute of Physics, Federal Research Center KSC SB RAS, Krasnoyarsk 660036, Russia; nico@iph.krasn.ru
2 School of Engineering and Construction, Siberian Federal University, Krasnoyarsk 660041, Russia;
3 Laboratory of Spectroscopy of Solid State, Ioffe Institute, St. Petersburg 194021, Russia; e.roginskii@mail.ioffe.ru
4 Laboratory of Radiospectroscopy and Spintronics, Kirensky Institute of Physics, Federal Research Center KSC SB RAS, Krasnoyarsk 660036, Russia; irinagudim@mail.ru (I.A.G.); bezm@iph.krasn.ru (V.L.T.)
5 Federal Research Center KSC SB RAS, Krasnoyarsk 660036, Russia; ivan_nemtsev@mail.ru
6 Laboratory of Crystal Physics, Kirensky Institute of Physics, Federal Research Center KSC SB RAS, Krasnoyarsk 660036, Russia; msmolokeev@mail.ru
7 School of Engineering Physics and Radio Electronics, Siberian Federal University, Krasnoyarsk 660041, Russia;
8 Institute of Automation and Electrometry, Russian Academy of Sciences, Novosibirsk 630090, Russia; adish2@ngs.ru (S.V.A.); apg@iae.nsk.su (A.M.P.)
9 Department of Inorganic and Physical Chemistry, Tyumen State University, Tyumen 625003, Russia; apg@iae.nsk.su
10 Department of General and Special Chemistry, Industrial University of Tyumen, Tyumen 625000, Russia
* Correspondence: oreshonkov@iph.krasn.ru

Received: 19 December 2019; Accepted: 21 January 2020; Published: 23 January 2020

Table S1. Fractional atomic coordinates and isotropic or equivalent isotropic displacement parameters (Å^2) of $YAl_3(BO_3)_4$ single crystal.

Atom	*x*	*y*	*z*	U_{iso}*/U_{eq}
Y	1.0000	1.0000	1.0000	0.0053 (3)
Al	0.44422 (18)	1.0000	1.0000	0.0060 (4)
B1	1.0000	1.0000	0.5000	0.0060 (13)*
B2	0.5570 (6)	1.0000	0.5000	0.0068 (10)*
O1	0.1494 (4)	1.0000	0.5000	0.0069 (7)
O2	0.4083 (4)	1.0000	0.5000	0.0086 (8)
O3	0.5506 (3)	0.8503 (3)	0.4794 (4)	0.0084 (5)

Table S2. The main bond lengths (Å) of $YAl_3(BO_3)_4$ single crystal.

Y—O3^{i}	2.318 (2)	B1—O1^{xiv}	1.387 (3)
Y—O3^{ii}	2.318 (2)	B1—O1^{xv}	1.387 (3)
Y—O3^{iii}	2.318 (2)	B1—O1^{xvi}	1.387 (3)
Y—O3^{iv}	2.318 (2)	B2—O3	1.369 (4)
Y—O3^{v}	2.318 (2)	B2—O3^{xvii}	1.369 (4)
Y—O3^{vi}	2.318 (2)	B2—O2	1.381 (7)
Al—O3^{x}	1.861 (3)	Al—O1^{xii}	1.916 (2)
Al—O3^{vii}	1.861 (3)	Al—O2^{xi}	1.927 (3)
Al—O1^{xi}	1.916 (2)	Al—O2^{xii}	1.927 (3)

Symmetry codes: (i) *y*+1/3, *x*+2/3, -*z*+5/3; (ii) -*x*+*y*+2/3, -*x*+4/3, *z*+1/3; (iii) -*y*+5/3, *x*-*y*+4/3, *z*+1/3; (iv) *x*+2/3, *y*+1/3, *z*+1/3; (v) -*x*+4/3, -*x*+*y*+2/3, -*z*+5/3; (vi) *x*-*y*+4/3, -*y*+5/3, -*z*+5/3; (vii) -*x*+*y*+1/3, -*x*+5/3, *z*+2/3; (viii) -*y*+7/3, *x*-*y*+5/3, *z*+2/3; (ix) *x*+1/3, *y*-1/3, *z*+2/3; (x) *y*-1/3, *x*+1/3, -*z*+4/3; (xi) -*y*+4/3, *x*-*y*+5/3, *z*+2/3; (xii) -*x*+*y*-1/3, -*x*+4/3, *z*+1/3; (xiii) -*y*+4/3, *x*-*y*+5/3, *z*-1/3; (xiv) -*x*+*y*, -*x*+1, *z*; (xv) *x*+1, *y*, *z*; (xvi) -*y*+2, *x*-*y*+2, *z*; (xvii) *x*-*y*+1, -*y*+2, -*z*+1

Table S3. Fractional atomic coordinates and isotropic displacement parameters (Å^2) of $YAl_3(BO_4)_3$ powder.

Atom	*x*	*y*	*z*	B_{iso}
Y	0	0	0	0.65 (7)
Al	0.5571 (2)	0	0	0.91 (9)
B1	0	0	0.5	1.7 (2)
B2	0.4432 (8)	0	0.5	0.86 (18)
O1	0.8497 (4)	0	0.5	1.07 (13)
O2	0.5928 (6)	0	0.5	1.10 (13)
O3	0.4490 (4)	0.1509 (4)	0.5196 (4)	0.74 (11)

Table S4. Main bond lengths (Å) of $YAl_3(BO_4)_3$ powder.

Y—O3^{i}	2.312 (3)	B1—O1^{iv}	1.396 (4)
Al—O1^{ii}	1.910 (3)	B2—O2	1.389 (9)
Al—O2^{ii}	1.912 (4)	B2—O3	1.382 (5)
Al—O3^{iii}	1.862 (3)		

Symmetry codes: (i) *y*-1/3, *x*-2/3, -*z*+1/3; (ii) -*y*+2/3, *x*-*y*-2/3, *z*-2/3; (iii) -*x*+*y*+2/3, -*x*+1/3, *z*-2/3

Table S5. Calculated optimized lattice parameters and atomic positions of $YAl_3(BO_3)_4$ in comparison with the experimental data.

	DFT			Exp.		
a, (Å)		9.0830			9.28485	
c, (Å)		6.9881			7.23005	
V, (Å^3)		499.28			539.79	
Y (3*a*)	0	0	0	0	0	0
Al (9*d*)	0.55782	0	0	0.5571	0	0
B1 (3*b*)	0	0	0.5	0	0	0.5
B2 (9*e*)	0.44134	0	0.5	0.4432	0	0.5
O1 (9*e*)	0.84888	0	0.5	0.8497	0	0.5
O2 (9*e*)	0.59178	0	0.5	0.5928	0	0.5
O3 (18*f*)	0.44639	0.14994	0.52520	0.4490	0.1509	0.5196

Table S6. Calculated and experimental phonon frequencies (cm^{-1}) of $YAl_3(BO_3)_4$ together with proposed assignments. Notations: ss – symmetric stretching, as – antisymmetric stretching, π – out-of-plane bending, δ – in-plane bending, libr. – librations, tr – translations.

**Calculated**				**Experimental**		**Assignment**
A_1	A_2	*E* (TO)	*E* (LO)	**Raman**	**Infrared**	
1327.10	1368.07	1381.59<br>1315.84<br>1268.23	1490.32<br>1344.37<br>1292.78	1453<br>1335<br>1314<br>1298<br>1287	1383<br>1348<br>1281<br>1254	BO_3 as
1039.74<br>959.24		1006.22	1008.16	1023<br>1015<br>982	984	BO_3 ss
	755.87	769.20	781.90	773<br>764	810<br>788<br>765	
697.94	692.47<br>640.88	701.75<br>668.32	702.40<br>668.38	714<br>705<br>690<br>673	724<br>705<br>675<br>662	BO_3 π and BO_3 δ
		613.95	642.29	646<br>620		
585.47		568.24	612.83	609<br>600	611	BO_3 δ + Al tr.
		497.28	535.53	555<br>527	577<br>535	
397.86	481.96<br>440.88<br>416.99<br>381.76	478.66<br>446.90<br>438.07<br>389.13	497.09<br>447.08<br>439.13<br>399.08	423<br>407<br>401<br>388	510<br>496<br>464<br>420	BO_3 t. + Al tr.
		351.52	354.36	344<br>338		BO_3 libr.
		334.21	334.65	307		
273.98	307.83<br>281.30<br>220.40	270.74<br>230.85	271.19<br>230.87	303<br>262<br>228		BO_3 libr. + Al tr.
		118.52	135.86	137<br>120		Y tr.
	63.83					Y tr.

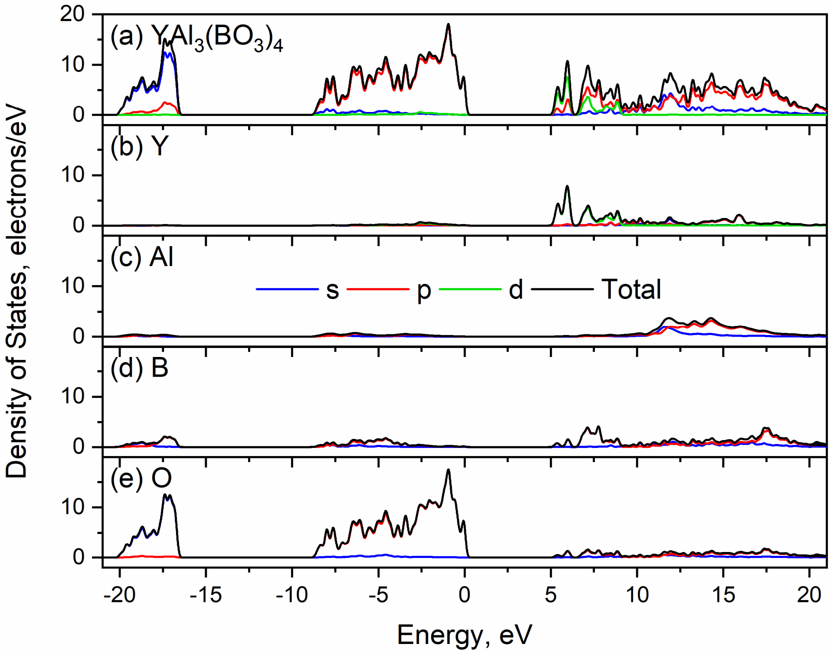

Figure S1. Total (**a**) and partial density of states (**b**), (**c**), (**d**), (**e**) of $YAl_3(BO_3)_4$.

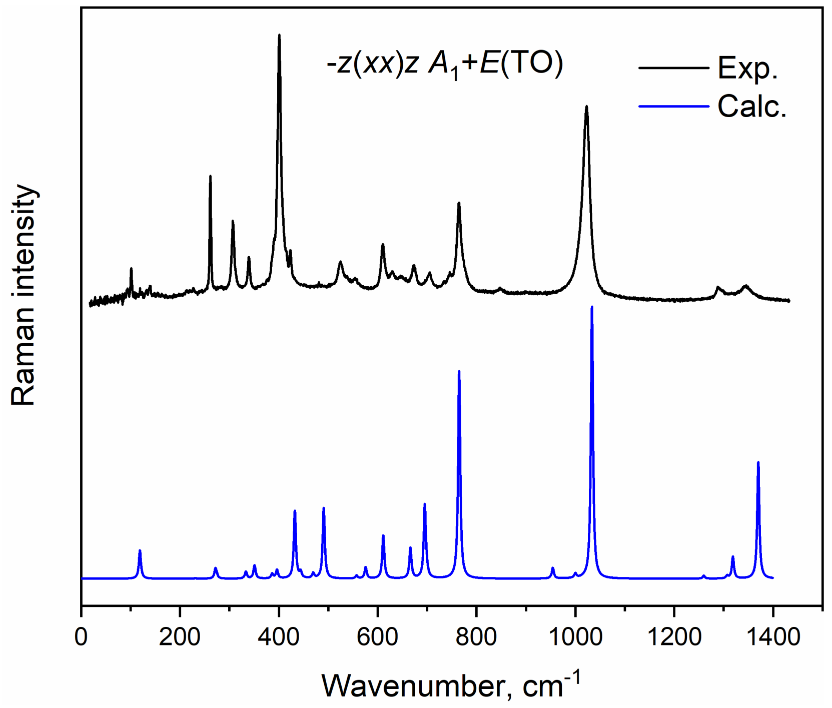

Figure 2. Polarized Raman spectrum of YAB single crystal obtained from the -*z*(*xx*)*z* orientation.

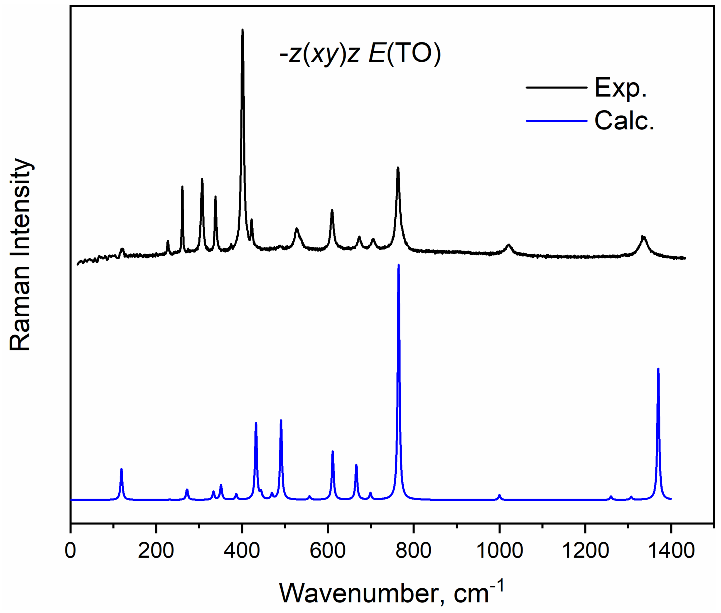

Figure S3. Polarized Raman spectrum of YAB single crystal obtained from the -*z*(*xy*)*z* orientation.

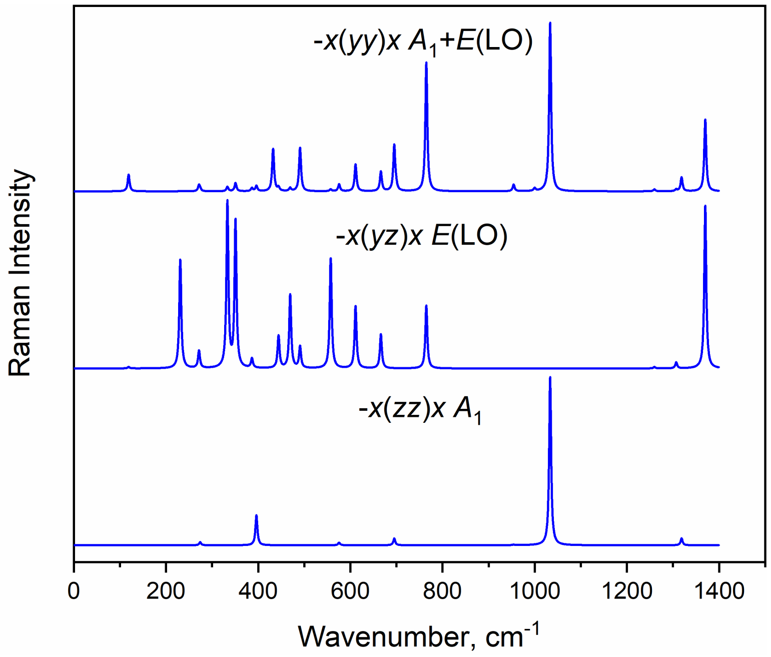

Figure S4. Calculated Raman spectra of YAB in the -*x*(*zz*)*x*, -*x*(*yz*)*x* and -*x*(*yy*)*x* polarizations.

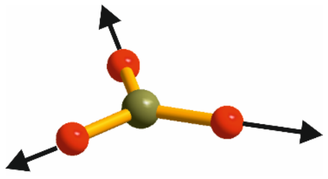

(**a**)

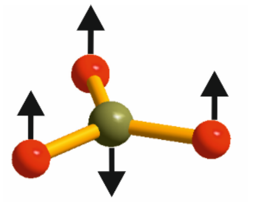

(**b**)

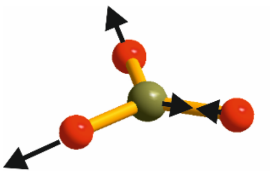

(**c**)

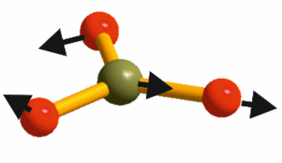

(**d**)

Figure 5. Normal modes of vibration of $[BO_3]^{3-}$ ions: (**a**) ν_1 symmetric stretching, (**b**) ν_2 out-of-plane bending, (**c**) ν_3 antisymmetric stretching, (**d**) v4 in-plane bending.

© 2020 by the authors. Licensee MDPI, Basel, Switzerland. This article is an open access article distributed under the terms and conditions of the Creative Commons Attribution (CC BY) license (http://creativecommons.org/licenses/by/4.0/).
